# Multicenter retrospective study of transcatheter arterial embolisation for life-threatening haemorrhage in patients with uncorrected bleeding diathesis

**DOI:** 10.1186/s42155-020-00186-3

**Published:** 2020-12-10

**Authors:** Stavros Spiliopoulos, Konstantinos Katsanos, Ioannis Paraskevopoulos, Martin Mariappan, Georgios Festas, Panagiotis Kitrou, Christos Papageorgiou, Lazaros Reppas, Konstantinos Palialexis, Dimitrios Karnabatidis, Elias Brountzos

**Affiliations:** 1 2nd Department of Radiology, Interventional Radiology Unit, Medical School, National and Kapodistrian University of Athens, “Attikon” University Hospital, Athens, Greece; 2 Department of Clinical Radiology, Interventional Radiology Unit, Aberdeen Royal Infirmary, NHS Grampian, Aberdeen, AB25 2ZN UK; 3 Department of Interventional Radiology, School of Medicine, Patras University Hospital, Rion, Greece

**Keywords:** Transcatheter arterial embolisation, Bleeding, Endovasdcular treatment, Coagulopathy, Prolonged clotting time

## Abstract

**Background:**

We retrospectively investigated outcomes of emergency TAE for the management of life-threatening haemorrhage in patients with uncorrected bleeding diathesis.

**Materials and methods:**

This multicenter, retrospective, study, was designed to investigate the safety and efficacy of percutaneous TAE for the management of life-threatening haemorrhage in patients with uncorrected bleeding disorder at the time of embolization. All consecutive patients with uncorrected coagulation who underwent TAE for the treatment of haemorrhage, between January 1st and December 31th 2019 in three European centers were included. Inclusion criteria were thrombocytopenia (platelet count < 50,000/mL) and/or International Normalized Ratio (INR) ≥2.0, and/or activated partial thromboplastin time (aPTT) > 45 s, and/or a pre-existing underlying blood-clotting disorder such as factor VIII, Von Willebrand disease, hepatic cirrhosis with abnormal liver function tests. Primary outcome measures were technical success, rebleeding rate and clinical success. Secondary outcome measures included patients’ 30-day survival rate, and procedure-related complications.

**Results:**

In total, 134 patients underwent TAE for bleeding control. A subgroup of 17 patients with 18 procedures [11 female, mean age 70.5 ± 15 years] which represent 12.7% of the total number of patients, presented with pathological coagulation parameters at the time of TAE (haemophilia *n* = 3, thrombocytopenia *n* = 1, cirrhosis *n* = 5, anticoagulants *n* = 7, secondary to bleeding *n* = 1) and were analyzed. Technical success was 100%, as in all procedures the bleeding site was detected and successfully embolised. Clinical success was 100%, as none of the patients died of bleeding during hospitalization, nor was surgically treated for bleeding relapse. Only one rebleeding case was noted (5.9%) that was successfully treated with a second TAE. No procedure-related complications were noted. According to Kaplan-Meier analysis the estimated 30-day survival rate was 84.2%.

**Conclusion:**

TAE in selected patients with uncorrected bleeding diathesis should be considered as a suitable individualized management approach. Emergency TAE for life threatening haemorrhage in patients with coagulation cascade disorders should be used as an aid in realistic clinical decision making.

## Introduction

Increasing experience and technological advances in embolic devices, coaxial catheter systems and microcatheters have established the role of percutaneous transcatheter arterial embolisation (TAE) in the modern treatment of haemorrhage (Rösch et al. 1972; Bookstein and Goldstein 1973; Bauer et al. 2004). TAE is today recommended in a variety of haemorrhagic conditions such as trauma, haemoptysis, tumors, etc. (Coccolini et al. 2017; Spiliopoulos et al. 2018; Chun et al. 2010; Revel-Mouroz et al. 2015; Chakraverty et al. 2012). Moreover, the continuously increasing population age, as well as the widespread use of more potent antithrombotic therapy has increased reports of severe spontaneous bleeding, while therapeutic anticoagulation at the time of bleeding, aggravates the severity of the event by triggering hemodynamic instability (Spiliopoulos et al. 2019). Additionally, underlying inherited or acquired coagulopathies, as well as acute traumatic coagulopathy and trauma induced coagulopathy, can exacerbate blood loss and delay treatment being independent predictors of mortality (Maegele 2019). Coagulopathies, either acquired (drug-induced, vitamin K deficiency, disseminated intravascular coagulation) or hereditary (hemophilia, von Willebrand disease), are not only correlated with an increased bleeding risk but have been also associated with clinical failure after transcatheter arterial embolization (Hur et al. 2014). Standard clinical practice in patients with clotting disorders includes correction of INR, aPPT and platelet values prior TAE, as this improves patient safety, increases clinical success and in several circumstances (mostly in spontaneous bleeding) incites haemostasis without further intervention (Spiliopoulos et al. 2018; Hur et al. 2014).

In 2012, the Society of Interventional Radiology (SIR), has issued a consensus document classifying endovascular procedures requiring sheath size up to 7Fr, as medium risk procedures and recommended that should be performed after optimization of the coagulation profile to the following values: INR < 1.5, aPTT < 1.5 x control and platelet count> 50.000/μL (Patel et al. 2012).

In 2019, SIR released two updated consensus documents recommending arterial puncture ≤6Fr and embolotherapy for patients presenting with INR values between 2 and 3, and PLTs < 50,000/μL, but this was based on an experts’ consensus rather than solid scientific evidence (Davidson et al. 2019; Pearl et al. 2019). Moreover, available consensus documents do not include specific recommendations for the management of bleeding diathesis prior, during, and after TAE for haemorrhage control, which represents a separate clinical setting, requiring rapid decision making and intervention, especially for cases in which time to optimize coagulation profile is minimal (Chakraverty et al. 2012).

In every day clinical practice, although deranged clotting is not an absolute contraindication for TAE, many interventional radiologists refuse TAE for patients with uncorrected bleeding parameters, until INR value normalizes around 1.5, driven by the lack of evidence regarding patient safety, in combination with the documented insufficiency of specific embolic materials, such as metallic coils, to provoke blood clotting in the presence of haemostatic disorders (Patel et al. 2012; Shi et al. 2017). Data regarding the safety and efficacy of TAE in patients with severe, life-threatening haemorrhage and bleeding diathesis due to anticoagulation therapy or underlying coagulopathy, remain scarce as they are mainly based on subgroups of patients with coagulopathy included in single-center, retrospective series (Hur et al. 2014; Shi et al. 2017; Jaffe et al. 2015; Baron et al. 2013). The authors sought to investigate outcomes of emergency TAE, performed in three large European vascular centers, for the management of life-threatening hemorrhage in patients with uncorrected bleeding diathesis.

## Materials and methods

**Fig. 1:**
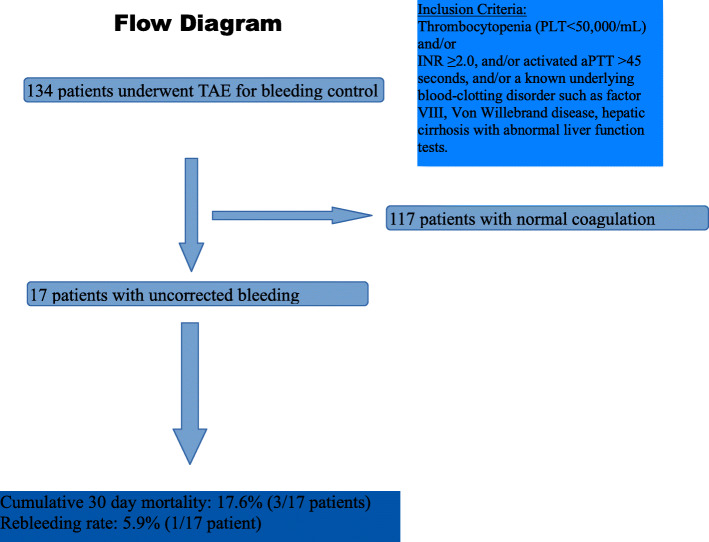
Study’s flow chart

This multicenter, retrospective, study, was designed to investigate the safety and efficacy of percutaneous TAE for the management of life-threatening haemorrhage in patients with uncorrected bleeding disorder at the time of embolization. Between January 1st and December 31th 2019 (one-year period), the electronic files all consecutive patients who underwent TAE in three European centers (A, P and A), for the treatment of haemorrhage, were searched and analysed. Institutional review board approval was not required for this retrospective study. A specific study informed consent was not necessary for inclusion in this retrospective study. A procedural written consent form has been obtained by all patients. Patients included in the analysis fulfilled the following criteria: (i) patients who underwent TAE due to haemorrhage within the predetermined 1-year period and (ii) uncorrected coagulation disorder at the time of embolization, defined as thrombocytopenia (platelet count < 50,000/mL) and/or International Normalized Ratio (INR) ≥2.0, and/or activated partial thromboplastin time (aPTT) > 45 s, and/or a known underlying blood-clotting disorder such as factor VIII, Von Willebrand disease, hepatic cirrhosis with abnormal liver function tests (Hur et al. 2014; Patel et al. 2012). The study’s flow chart is depicted in Fig. 1. In all participating centres, TAE was decided following a multidisciplinary team (MDT) decision involving the treating physician, the surgical team and the hematology team. The MDT organized persistent efforts to improve the coagulation profile prior, during and after embolization in all patients. The MDT decision to timely proceed to emergency embolization in patients with uncorrected bleeding diathesis was balanced and TAE was offered in cases in which patient survival was compromised by ongoing life-threatening bleeding leaving no available time for further improvement of the coagulation parameters. TAE was performed using standard interventional techniques according to international guidelines (Chakraverty et al. 2012). In brief, common femoral artery access was used and microcatheters were used (Progreat 2.7Fr; TERUMO or Masters 2.6Fr; ASHAHI INTECC, CO) for super-selective catheterization of the bleeding branches whenever necessary. The choice of embolic materials was case-sensitive and decided by the operator according to his experience, the embolization goal and the medical centre’s device availability.

### Statistics

Continuous data regarding rebleeding rate were evaluated with the Kolmogorov-Smirnov goodness-of-fit test to determine whether they were originating from normal distributions and the Mann-Whitney test was performed for the comparison of rebleeding rate between the two cohorts of deranged and normal coagulation profiles, as variables did not pass the normality test. Patients’ 30-day survival was estimated using Kaplan-Meier analysis. Statistical analysis was performed with SPSS statistical software (version 25, IBM).

### Endpoints and definitions

The primary outcome measures were technical success, defined as occlusion of bleeding site(s) with no signs of bleeding at completion DSA, recurrent haemorrhage rate defined as relapse of bleeding at the same site following successful TAE, re-intervention rate due to recurrent haemorrhage and clinical success defined as the absence of bleeding related death during hospitalization. Secondary outcome measures were patients’ 30-day survival rate, rebleeding rate compared to that of patients without deranged coagulation at the time of embolization, and procedure-related complications, classified according to the CIRSE 6-grade classification system (Filippiadis et al. 2017). All primary and secondary outcomes were statistically analysed on a per patient basis.

## Results

In total, 134 patients underwent TAE for bleeding control within the study’s one-year time period and 12.7% [17 patients; 11 female (64.7%)] with a mean age 70.5 ± 15 years (range: 23–87), presented with pathological coagulation parameters indicating increased bleeding diathesis at the time of TAE and were enrolled in the study. All 17 patients underwent computed tomography angiography (CTA) which was positive for active bleeding prior the procedure. In total 18 TAE procedures were performed and analysed. In one patient underlying coagulopathy (congenital haemophilia) was not known at the time of embolization and all other bleeding parameters were within normal range. INR ≥2 was noted in seven patients (two with concomitant prolonged aPTT > 45). Prolonged aPTT > 45 was observed in five patients. Underlying Coagulopathy was recorded in seven patients and included three cases of haemophilia (two cases of congenital Von Willebrand disease and one case of acquired factor VIII deficiency), one case of thrombocytopenia (PLTs 29 K/μL) and five cases of cirrhosis with severe total protein and albumin deficit (three cases of decompensated cirrhosis). In three patients with underlying coagulopathy, INR values ≥1.7 were recorded (two with cirrhosis and one with acquired factor VIII deficiency). Patients’ baseline demographical and clinical-laboratory data, as well as procedural details are analytically presented in Table 1.

**Table 1:** Demographical data, coagulation data and procedural details

Pt	Gender	Age (years)	Underlying pathology/anticoagulation	Coagulation profile	Cause	Bleeding vessel	Embolization material	Sheath size/removed	Closure device	Rebleeding
1	Female	75	Warfarin	INR 2.2; PT 19.2; aPTT: 27.8; PLT: 258 K/μL	Spontaneous	Lt Subclavian artery branch	Pushable microcoils (×2) and 300-500 μm microparticles	6 Fr/same day	Angioseal	No
2	Male	83	Warfarin	INR 2.14; PT 20.3; aPTT: 37.4; PLT: 184 K/μL	Trauma	Right renal artery branch	Gelfoam and pushable microcoils (×3)	6 Fr/same day	Perclose	No
3	Female	70	Warfarin	INR 4.3; PT 41.1; aPTT: 33.1; PLT: 141 K/μL	Spontaneous	Right inferior epigastric artery	Glue 1:3	6 Fr/same day	Starclose	No
4	Female	82	Giant AML (16 cm) rupture	INR 3.5; PT 20.3; aPTT: 37.4; PLT: 184 K/μL	Trauma	Left renal artery branches	Microspheres 300–500 and 500–700 and microcoils (×3)	6 Fr/24 h	Starclose	No
5	Female	87	Acquired factor VIII deficiency/ LMWH 40 mg twice daily	INR 1.8; PT 21.5; aPTT: 164.1; PLT: 483 K/μL	Spontaneous	Right deep femoral artery branches	Gelfoam	6 Fr/same day	Starclose	Yes; successfully treated with 3 microcoils and gelfoam
6	Female	23	Congenital hemophilia	INR 1.37; PT 14.3; aPTT: 30.5; PLT: 141 K/μL	Iatrogenic: post renal biopsy	Right renal artery branch	Glue (1:1) and pushable microcoil (×1)	6 Fr/same day	Angioseal	No
7	Female	74	Decompensated cirrhosis: Alk phos 50 U/L; Albumin 12 g/L; total proteins 21 g/L, Bilirubin 22 umol/L	INR 1.0; PT 12.1; aPTT: 29.8; PLT: 55 K/μL	Iatrogenic: post-liver biopsy	Right hepatic artery	Microparticles 500-700 μm and gelfoam	6 Fr/same day	Starclose	No
8	Female	72	Decompensated cirrhosis/gastric varices: Alk phos 67 U/L; Albumin 19 g/L; total proteins 31 g/L, Bilirubin 54 umol/L	INR 4.5; PT 46; aPTT: 124.2; PLT: 348 K/μL	Spontaneous	Left hepatic artery	Microparticles 300–500 μm and pushable microcoils (×3)	6 Fr/same day	Starclose	No
9	Female	68	LMWH 60 mg once daily	INR 2.0; PT 26.2; aPTT: 50.7; PLT: 210 K/μL	Trauma	Right internal iliac artery	Pushable microcoils (×9)	6 Fr/same day	Starclose	No
10	Female	75	LMWH 40 mg twice daily	INR 1.2; PT 17.5; aPTT: 121.3; PLT: 176 K/μL	Spontaneous	DFA branch	Pushable microcoils (×2)	6 Fr/same day	Starclose	No
11	Male	64	Warfarin	INR 4.7; PT: 74.1; aPTT: 48.3; PLT: 348 K/μL	Spontaneous	SMA branch	Pushable microcoils (×7)	6 Fr/24 h	Angioseal	No
12	Female	86	LMWH 40 mg twice daily	INR 1.2; PT: 17.0; aPTT: 95.3; PLT: 205 K/μL	Spontaneous	Left inferior epigastric artery and left deep femoral artery branches	Onyx Ethylene vinyl alcohol copolymer (EVOH), Microparticles 300-500 μm and pushable microcoils (×5)	6 Fr/same day	Starclose	No
13	Male	59	Thrombocytopenia	INR 1.2; PT: 12.7; aPTT: 36; PLT: 29 K/μL;	Spontaneous	Left 3rd Lumbar artery	Onyx Ethylene vinyl alcohol copolymer (EVOH)	6 Fr/72 h	Angioseal	No
14	Male	65	Congenital hemophilia	INR 1.1; PT 12.0; aPTT: 29.3; PLT: 152 K/μL	Iatrogenic: post-arterial access	Right deep femoral artery pseudoaneurysm	Glue 1:1 and pushable microcoil (×1)	6 Fr/same day	Angioseal	No
15	Male	66	Cirrhosis; Alk phos 67 U/L; Albumin 21 g/L; total proteins 63 g/L, Bilirubin 228 umol/L	INR 1.7; PT: 20; aPTT: 39.4; PLT: 59 K/μL	Spontaneous	Left inferior epigastric artery	Gelfoam and pushable microcoils (×2)	6 Fr/same day	Starclose	No
16	Male	82	Decompensated cirrhosis/ jaundice: Alk phos 227 U/L; Albumin 17 g/L; Total protein 44 g/L; AAT 127 U/L; GGT 211 U/L; Bilirubin 217umol/L	INR 1.0; PT: 10.6; aPTT: 32.9; PLT: 185 K/μL	Spontaneous	Left gastric artery	Pushable microcoils (×4)	6 Fr/same day	Angioseal	No
17	Female	69	Cirrhosis:: Alk phos 114 U/L; Albumin 31 g/L; Total protein 54 g/L; AAT 44 U/L; GGT 114 U/L; Bilirubin 24umol/L	INR 1.7; PT: 19.3; aPTT: 26.7; PLT: 166 K/μL	Spontaneous	Right hepatic artery	Microparticles 300-500 μm, detachable microcoil (×1), gelfoam	6 Fr/same day	Angioseal	No

Glue used: N-butyl-2 cyanoacrylate (NBCA)

Microparticles used: Embozene microspheres; CELONOVA and Embosphere® Microspheres; MERIT Medical

Normal laboratory value range: PT: 10–12 s, aPTT: 30–45 s PLT: 150–400 K/μL INR: 0.80–1.5

Compication rate: 5,9% (1/17)

In most of the cases liquid embolic material was preferred such as N-butyl cyanoacrylate (Gluebran®, GEM SRL) in three cases; Onyx® (Medtronic) in two cases and gelfoam (Gelfoam; Pfizer or Embocube 2.5 mm; MERIT Medical) in six cases (11/18 procedures; 61.1%), either alone (3/18 procedures; 16.6%) or in combination with other embolic materials such as microparticles (Embozene microspheres; CELONOVA or Embosphere® Microspheres; MERIT Medical) or microcoils (pushable Nestercoils; COOK, or CONCERTO™ Helix detachable microcoils; Medtronic) or both (Table 1). In four procedures only microcoils were used (4/18; 22.2%).

**Fig. 2:**
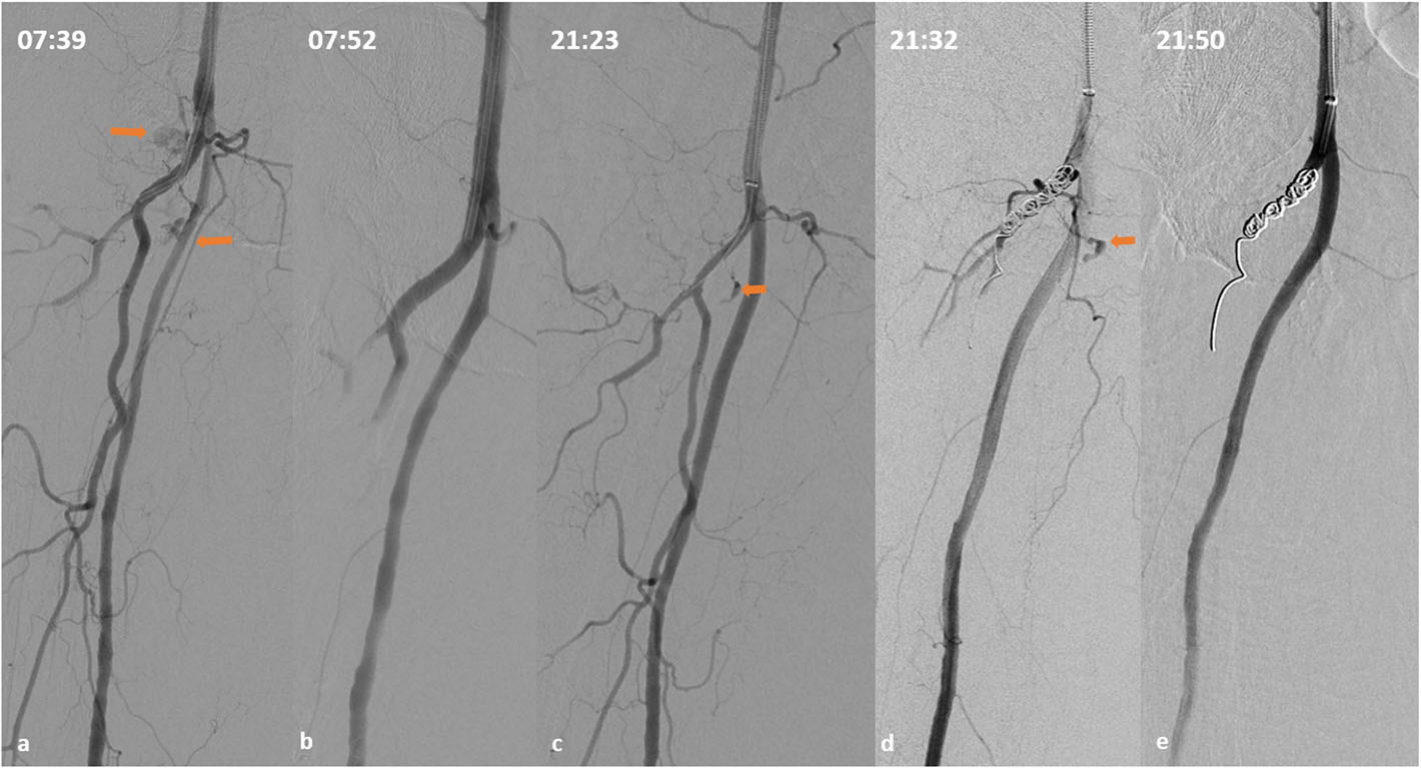
The single case of rebleeding. An 87-year old female patient with acquired factor VIII deficit, aPTT 164.1; INR 1.8 and spontaneous bleeding of a right deep femoral artery branch, causing significant Ht/Hb decrease and hemodynamic instability. **a** Selective DSA demonstrating two sites of active extravasation from branches of the DFA. **b** Final DSA following gel foam embolization. **c** Selective DSA approx. 13 h after first embolization demonstrating rebleeding from one of the previously embolized bleeding sites (arrow). aPTT is now prolonged. **d** Persisting active bleeding through the coils, approx 5 min after their deployment. **e** Final DSA demonstrating successful hemostasis following further embolization with gel foam after placing the microcatheter within the coils

Technical success was 100%, as in all procedures the bleeding site was detected and embolized, without angiographical signs of haemorrhage at final DSA. Clinical success was 100%, as none of the patients died of bleeding during hospitalization, nor was surgically treated for bleeding relapse. In all 18 procedures 6Fr sheaths were used and a closure device was deployed to achieve haemostasis in all cases (seven Angio-Seal™; TERUMO Europe, Belgium; ten Starclose SE™ and one Perclose ProGlide™; ABBOTT, USA). No access-site bleeding complications were noted, apart from access-related soft, small groin haematomas. Rebleeding occurred in one patient (rebleeding rate 5.9%; 1/17 cases). The single rebleeding episode occurred in an 87-year old female patient with acquired factor VIII deficiency (attributed to pneumonia), heparin therapy for venous thrombosis (enoxaparin 40 mg twice daily), and spontaneous active bleeding from a right deep femoral artery DFA branch causing enlarging thigh haematoma. Factor VIII was not readily available at the hospital and was awaited. Hemoglobin (Hb) dropped from 8.5 g/dL at arrival to 7.5 g/dL in 2 h and further decreased to 5.1 g/dL at the time of the first embolization, despite transfusions (2 bags of plasma 2 units of RBCs and transamine administration). The patient was unstable (systolic blood pressure (SBP): 65 mmHg under inotropic therapy] INR was 1.8 and aPTT was 164.1 at the time of the first technically successful embolization using gel foam (Fig. 2a-b). The patient was immediately stabilized (SBP 100 mmHg without inotropic support) and Hb increased to 7.8 g/dL. However, after 12 h Hb dropped to 6.5 g/dL and aPTT was again indefinable. The patient was now haemodynamically stable. CTA detected rebleeding at the same location. DSA confirmed bleeding from the previously embolized DFA branch (Fig. 2c) and reintervention using further foam and coiling of the origin of the DFA was successfully performed (Fig. 2d). The patient received factor VIII approximately 8 h after the second embolization and no further bleeding was noted. She died after 17 days in ICU due to respiratory insufficiency caused by pneumonia. Rebleeding rate was similar compared to the cohort of patients embolized during the same time period [5.9% (1/17 cases) versus 4.3% (5/117 cases), *p* = 0.38; respectively]. No procedure-related complications were noted. In three cases (3/18 procedures; 16.6%) the performing physician decided to remove the arterial sheath at approximately 24 and 72 h following the procedureIn all cases a closure device was used for haemostasis, without any sequalae. The day following TAE, normalization of the coagulation profile (INR ≤ 1.7 and aPTT< 45 and platelet count > 50,000/mL) was noted in 7/12 cases (58.3%) with previously abnormal INR and/or aPTT and/or PLT count.

**Fig. 3:**
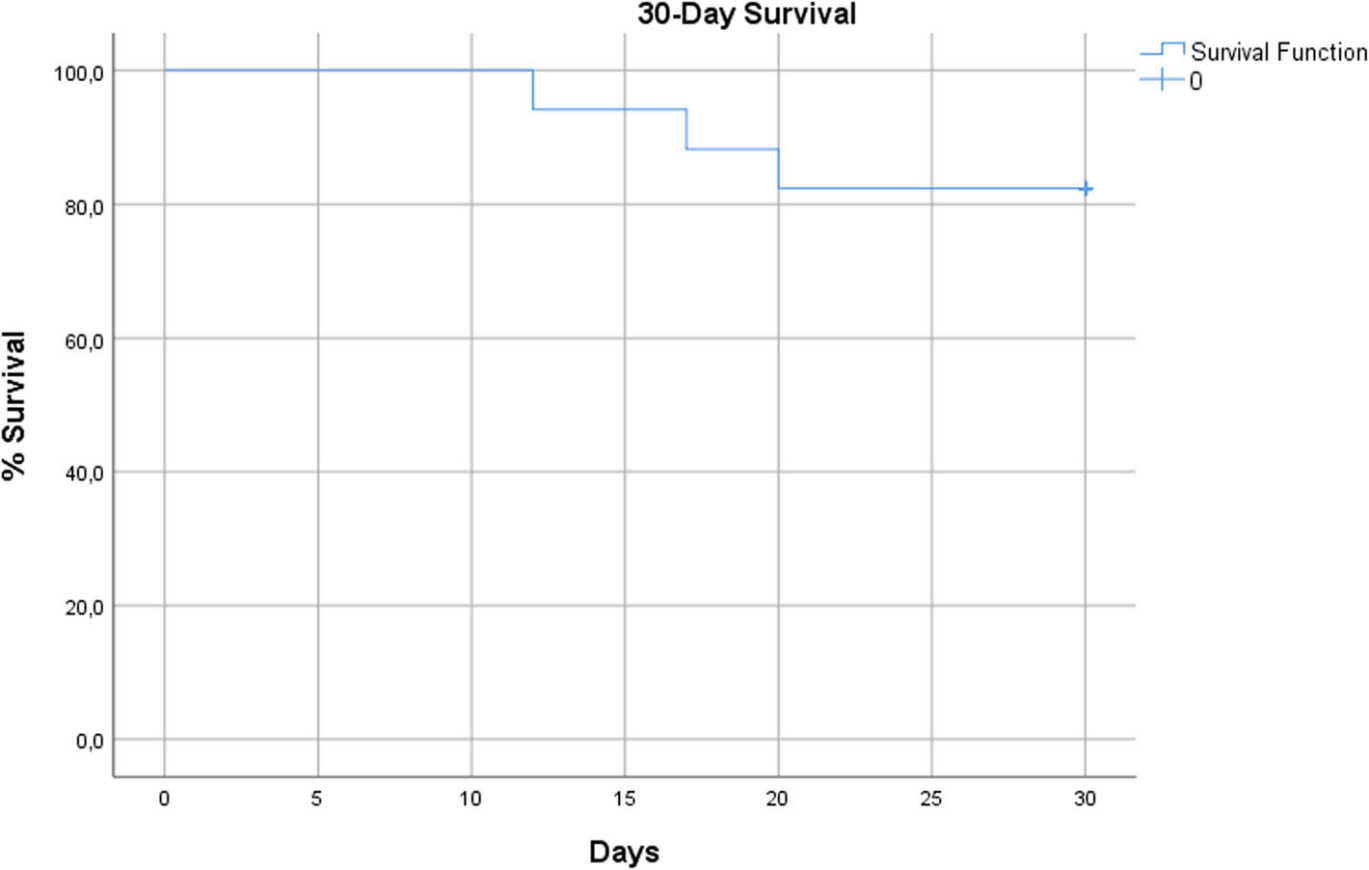
Kaplan-Meier plot of 30-day survival rate

In total, three deaths were recorded during hospitalization and the cumulative 30-day mortality rate was 17.6% (3/17 cases). According to Kaplan-Meier analysis the estimated 30-day survival rate was 84.2% (Fig. 3). Death rate directly related to bleeding was zero, as two patients died of pneumonia in ICU 17 days and 20 days after TAE (patients #5 and #11 in Table 1) and one due to cholangitis 12 days after TAE (patient #16 in Table 1).

## Discussion

The annual incidence of TAE procedures performed in the three participating tertiary European vascular centres for the management of severe haemorrhage in patients with uncorrected bleeding diathesis, was surprisingly high (12.7%; approximately one out of ten patients who underwent TAE in 2019). The results of this retrospective, real-life, study, demonstrated that TAE in the specific cases was safe and effective and could increase the therapeutic window in carefully selected patients. Specifically, technical success was 100% and only one case of rebleeding/repeat TAE was recorded that was successfully treated with further TAE. The clinical safety and effectiveness of TAE has been widely reported in the literature, establishing its role in bleeding control (Spiliopoulos et al. 2019; Hur et al. 2014; Melloul et al. 2015; Padia et al. 2020). However, uncorrected coagulopathy related to anticoagulation, haemophilia, cirrhosis or severe trauma is a relative contraindication for TAE, as it has been previously reported as a significant negative predictor for recurrent bleeding, in-hospital mortality and procedure-related complication rates, up to 3.5 times more likely to result in clinical failure (Hur et al. 2014; Valek and Husty 2013; Angle et al. 2010; Loffroy et al. 2010; Lopera 2010). Loffroy et al., have reported that patients with impaired coagulation demonstrate three times higher risk for rebleeding following successful TAE and ten times higher bleeding-related risk of death compared to patients with normal coagulation profile. Nevertheless, the authors highlighted the fact that these patients should not be refused emergency TAE while efforts to correct coagulation before, during, and after embolization, are of most importance for definite clinical success (Loffroy et al. 2010). This study reports similar technical success and rebleeding rates between patients with severe bleeding diathesis and those with normal coagulation profile at the time of embolization. Moreover, no procedure-related complications were noted, while the 30-day survival rate was considered extremely satisfactory, given the advanced age and multiple comorbidities noted in the specific cohort.

The authors speculate that the above-mentioned outcomes could be attributed to several factors, among which are the long-standing local experience and expertise in the multi-disciplinary management of haemorrhage, that includes optimal periprocedural coagulation improvement, as well as specific considerations regarding the timing and technique of TAE. Notably, efforts to improve the coagulation profile prior, during and after embolization were made in all patients and in 58% of the cases, abnormal INR, aPTT and PLT values were normalized within 24 h, a fact that certainly contributed to the minimal rebleeding rate and overall clinical success. The decision to timely proceed to emergency embolization in patients with uncorrected bleeding diathesis was balanced between the risk of technical failure/procedure-related complications and patient survival, which was severely compromised by ongoing bleeding causing hemodynamic instability, leaving no time for further improvement of the coagulation parameters. Such emergency multidisciplinary clinical decisions are currently supported by international experts’ consensus (Patel et al. 2012). Of note, time to embolization has been correlated with better clinical outcomes and waiting for a long time period in order to normalize clotting time is not a good option in cases of large-volume bleeding causing hemodynamic instability (Chakraverty et al. 2012).

In this study, the choice of embolic material was heterogeneous and highly individualized, a fact that should be expected in a retrospective analysis. It is well-known that correct choice of the embolization agents is essential. However, in patients with impaired coagulation, the choice of embolic agents is even more crucial and should be based on unique particularities. The use of metallic coils has been correlated with ineffective haemostasis, as their mechanism of action is based on the organism’s intrinsic ability to form clot. This is less likely to occur when the coagulation cascade has been impaired. In this series, blood flow through the coils has been noted even 5 min after coil deployment in a patient with factor VIII deficiency and an undefinable aPTT value (Fig. 1). The same applies for gelfoam as its mechanism of action also involves mechanical obstruction, blood flow reduction and therefore thrombus formation. However, dense coil packing or a combination of dense coiling with gelfoam could provoke complete arterial blockage and successful hemostasis (Fig. 1). Solely dense coil embolization was successfully performed in four cases, without any bleeding recurrence. Although, not commonly preferred, successful coil-only embolization has been previously reported for patients with significantly prolonged INR (> 3) (Ramaswamy 2014). Choosing to use only coils, is usually based on the safety provided by coiling in order to avoid non-targeted embolization and severe ischemic complications, such as in cases in which selective catheterization is not possible or for lesions close to arterial origins and bifurcations (Fig. 1), where glue or particles could easily migrate. It is important to understand that correct dense coil packing can be efficient even in cases of deranged clotting, especially if prompt normalization of the coagulation profile is awaited shortly after TAE. Microparticles, also act as blood-flow blockers, but their mechanism of action also involves an immediate inflammatory reaction of the arterial wall, focal angionecrosis and eventual vessel fibrosis and theoretically should be less influenced by deranged coagulation. In this study, microparticles were successfully used but only in combination with microcoils, and generally various combination of embolic agents was used in most cases (in order to enhance the occlusive effect, as previously described (Aina et al. 2001). On the other hand, n-butyl cyanoacrylate glue and ethylene vinyl alcohol copolymer (Onyx®) have been proposed as the most effective embolic agents in patients with bleeding disorders, as polymerization (glue) and solidification (Onyx) abruptly occludes the target vessel independently from the coagulation process (Shi et al. 2017; Toyoda et al. 1996; Né et al. 2018).

In the single case of rebleeding, gel foam was used a spontaneous bleeding originating from a DFA branch, in a patient with acquired factor VIII deficit and hemodynamic instability. Re-embolization using a combination of microcoils and gel foam was successfully performed, and bleeding control was achieved following concomitant factor VIII administration, which was not available at the time of initial embolization. Despite bleeding relapse, the first TAE procedure, contributed in stabilising the patient hemodynamically. The second hemorrhagic event occurred after 12 h. Subsequent second successful TAE in combination with factor VIII administration resulted in successful haemostasis.

Another important issue is access-site haemostasis and related complications. Manual compression in patients with prolonged coagulation time can be extremely long-lasting, tedious and ineffective. A closure device was used in all patients and no access-site complications were noted. In two cases the sheath was removed the next day and in one case after approximately 72 h. Choosing not to remove the sheath immediately after the procedure is always a choice if abnormal coagulation parameters are expected to normalize or significantly improve over time. Smaller 4Fr or 5Fr sheaths would be an option in these high bleeding risk cohort, but hemostasis should be performed using manual compression, as available closure devices use 6Fr sheaths. The authors preferred the use of a closure device over manual compression for smaller access diameters in the specific cohort, although this is not supported by evidence.

Limitations of this study include the retrospective design which could generate a selection bias, while some cases and data could have been missed. Moreover, cases that were discussed by the MDT for possible TAE, but finally underwent endoscopy or surgery, could not be identified. Additionally, this was not designed to be a comparative study with a control group and comparative data of rebleeding rate with the group of patients demonstrating normal coagulation profile could be confounded by significant heterogeneity. Notably, additional conservative treatments given to patients (for example cryoprecipitate) may have increased procedural and clinical success, but study design did not allow for the assessment of the contribution of such factors to the study outcome measures. Finally, the relatively small number of patients included, limits the validity of outcomes.

## Conclusions

This study provides valuable data supporting the option of life-saving TAE for haemorrhage control in selected patients with uncorrected bleeding diathesis, in order to avoid unnecessary delays while trying to correct coagulation parameters that either cannot be corrected or do not require correction for emergency TAE.

## Data Availability

The datasets used and/or analysed during the current study are available from the corresponding author on reasonable request.

## Abbreviations

aPTT: Activated Partial Thromboplastin Time
DFA: Deep Femoral Artery
DSA: Digital Subtraction Angiography
Hb: Hemoglobin
Ht: Hematocrit
ICU: Intensive Care Unit
INR: International Normalized Ratio
PLT: Platelets
PT: Prothrombin time
TAE: Transcatheter Arterial Embolisation
